# A REVIEW TO HONOR THE HISTORICAL CONTRIBUTIONS OF PAULINE GROSS, ALDRED WARTHIN, AND HENRY LYNCH IN THE DESCRIPTION AND RECOGNITION OF INHERITANCE IN COLORECTAL CANCER

**DOI:** 10.1590/0102-6720202400019e1812

**Published:** 2024-07-01

**Authors:** Fábio Guilherme CAMPOS, Leonardo Afonso BUSTAMANTE-LOPEZ, Luiz Augusto Carneiro D’ALBUQUERQUE, Ulysses RIBEIRO, Paulo HERMAN, Carlos Augusto Real MARTINEZ

**Affiliations:** 1 Universidade de São Paulo, Faculty of Medicine, Gastroenterology Department, Colorectal Surgery Division – São Paulo (SP), Brazil;; 2 AdventHealth Central Florida, Research Fellow of Colorectal Surgery – Sanford, Florida, USA;; 3 Universidade de São Paulo, Faculty of Medicine, Gastroenterology Department, Hepatic and Digestive Organs Transplant Division – São Paulo (SP), Brazil;; 4 Universidade de São Paulo, Faculty of Medicine, Gastroenterology Department, Gastric and Small Bowel Division – São Paulo (SP), Brazil;; 5 Universidade de São Paulo, Faculty of Medicine, Gastroenterology Department, Hepatic Surgery Division – São Paulo (SP), Brazil;; 6 Universidade Estadual de Campinas, Faculty of Medical Sciences, Department of Surgery, Colorectal Division – Campinas (SP), Brazil;; 7 Universidade São Francisco, Faculty of Medicine – Bragança Paulista (SP), Brazil.

**Keywords:** Lynch Syndrome, Hereditary Nonpolyposis Colorectal Neoplasms, Ancient Histories (Medicine), Biography., Síndrome de Lynch, Neoplasia Colorretal Hereditária não Polipoide, Histórias Antigas (Medicina), Biografia.

## Abstract

The present manuscript aimed to review the historical development and most important contributions regarding Lynch Syndrome since its first description, more than a century ago. In 1895, a reputed pathologist from Michigan University, Dr. Aldred Scott Warthin, got intrigued by the family history of a local seamstress called Pauline Gross. According to her prevision, she would present an early death due to cancer, which actually happened (from the uterus). Historically, her family was designated “Family G”, comprising a group recognized as the longest and most detailed cancer genealogy that has ever been studied. Warthin concluded that its members had genetic susceptibility for cancer, and they are, nowadays, considered the first reported Lynch Syndrome family. At that time, however, the medical cancer community was far less receptive to the association between heredity and cancer, despite the description of other families with similar heredograms. Unfortunately, this historical fact remained somewhat dormant until another investigator inaugurated a new era in the understanding of family cancer clusters. After reports and studies from this family and many others, the condition initially called Cancer Family Syndrome was changed to the eponym Lynch Syndrome. This was a recognition of the extensive and dedicated work developed by Dr. Henry Lynch in describing various characteristics of the disease, and his efforts to establish the correct recommendations for its diagnosis and treatment. Although the future announces there is still far to go for a complete understanding of Lynch Syndrome, the remarkable contributions of Pauline’s intuition, Warthin’s perseverance, and Lynch’s work consistency must never be forgotten by those who already have or will still benefit from this knowledge.

## RESUMO

O objetivo do presente manuscrito foi fazer uma revisão histórica do desenvolvimento e das mais importantes contribuições em relação à Síndrome de Lynch, desde sua primeira descrição há mais de um século atrás. Em 1895, o reputado patologista Dr. Aldred Scott Warthin ficou intrigado com a história familiar de uma costureira local, chamada Pauline Gross. De acordo com a sua previsão, ela morreria precocemente devido a um câncer, o que realmente aconteceu (do útero). Historicamente, sua família foi designada como Família “G”, caracterizando um grupo reconhecido como a maior e mais longa árvore genealógica relacionada ao câncer familiar jamais estudada. Warthin concluiu que os membros dessa família tinham susceptibilidade genética para câncer, e ainda hoje são considerados a primeira família com Síndrome de Lynch reportada na literatura. Entretanto, naquela época a comunidade médica oncológica não era receptiva à associação entre hereditariedade e câncer, a despeito da descrição de outras famílias com heredogramas similares. Infelizmente, esse fato histórico permaneceu esquecido até que outro investigador inaugurou uma nova era para a melhor compreensão da agregação familiar do câncer. Após diversas descrições dessa mesma agregação de casos de câncer em outras famílias, essa condição inicialmente denominada Síndrome de Câncer Familial foi mudada para o epônimo Síndrome de Lynch. Esse foi um reconhecimento ao extenso e dedicado trabalho desenvolvido pelo Dr. Henry Lynch na descrição de diversas características da doença e seus esforços para estabelecer as recomendações corretas para o seu diagnóstico e tratamento. Embora o futuro anuncie que ainda teremos um longo caminho a percorrer para a completa compreensão da Síndrome de Lynch, as contribuições extraordinárias da intuição de Pauline, da perseverança de Warthin e da consistência do trabalho de Lynch nunca devem ser esquecidas por àqueles que já se beneficiaram, bem como os que ainda irão se beneficiar de todo esse conhecimento.

## INTRODUCTION

During the 19^th^ century, colorectal cancer (CRC) etiology was associated only with environmental factors. Many famous investigators such as Theodore Billroth (mucosal origin), Karl Heinrich Bauer (sudden mutation in somatic cell), and Cuthbert Esquire Dukes (benign mucosal polyps) defended different ideas and possible origins for this neoplasm, and the subject persisted as a matter of intense debate^9^.

In recent decades, the comprehension of molecular mechanisms involved in CRC carcinogenesis has experienced a great improvement thanks to the work of notable investigators^16^. In this setting, identification of patients harboring mutations implicated in hereditary syndromes is crucial, with significant impact on cancer prevention, clinical decision-making, and surveillance^39^.

This is especially true for Lynch Syndrome (LS) patients. It is one of the first hereditary cancer-prone syndromes to be recognized and also the most prevalent^39^. These mutations are responsible for genome integrity and can predispose to many types of cancers, especially CRC and endometrial tumors.

The present manuscript aimed to review some historical aspects and interesting details concerning the first description of a family with LS more than a century ago. With this, we also want to pay tribute to those who contributed to all the knowledge we share today about this subject.

### The early days of 20^th^ century: the intuition of Pauline Gross and the perseverance of Dr. Aldred Warthin

**Figure 1:**
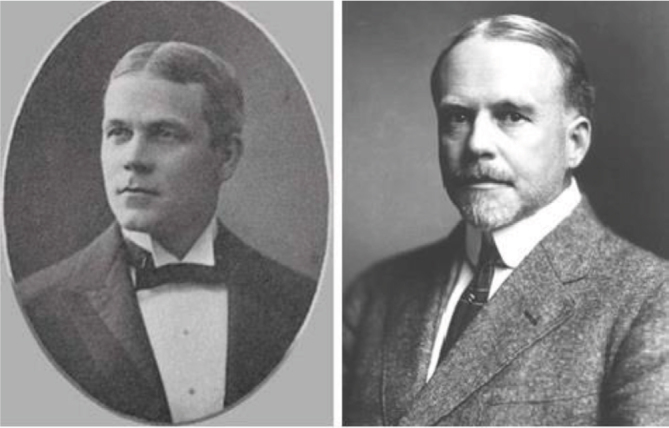
Aldred Scott Warthin (1866–1931), Director of the Pathological Laboratory at Michigan University. A photo as a young man (around 1900, left) and some years later (around 1925, right). (Left: Available from: http://www.homeoint.org/cazalet/histo/michigan.htm; Right: Available from: https://pt.findagrave.com/memorial/51252820/aldred-scott-warthin).

The first personage in the evolution of hereditary CRC was a young doctor named Aldred Scott Warthin (Figure 1, left). He was born on October 21, 1866, in Greensburg, Indiana. As a young man, he studied piano and earned a teacher’s diploma from the Cincinnati Conservatory of Music in 1877^26^. Warthin received an Arts Master’s degree (AM) in 1890, a Doctor of Medicine’s (MD) degree in 1891, and a PhD degree in 1893 from the University of Michigan in Ann Arbor^51^. While in medical school, he supported himself by teaching music and working as a church organist^51^. He did postgraduate study in Vienna and Freiburg and was designated as a demonstrator in pathology at the University of Michigan after returning from Europe in 1895. He was promoted to Assistant Professor in 1899, Junior Professor in 1902, and Full Professor in 1903, when he was also appointed Director of the Pathological Laboratory in Ann Arbor. He held these positions until his death in 1931 (Figure 1, right)^42,51^.

One day, while returning home from work, he met his family’s young seamstress, called Pauline Gross. He became deeply intrigued with the problem that afflicted her as she seemed very depressed over the thought she would undoubtedly die of any malignancy, because most of her relatives had precocious deaths associated with gastric, colorectal, or gynecological cancer^40^. She told Warthin: *“I’m healthy now, but I fully expect to die an early death”*.

In fact, Pauline’s grim was not mere pessimism. She believed her fate was sealed because her grandmother and grandfather who came from *Plattenhardt* (Germany) in the 1830s and many of their descendants suffered from cancer through generations^6^. According to her prevision, she actually died at an early age due to metastatic endometrial cancer^26^.

**Figure 2:**
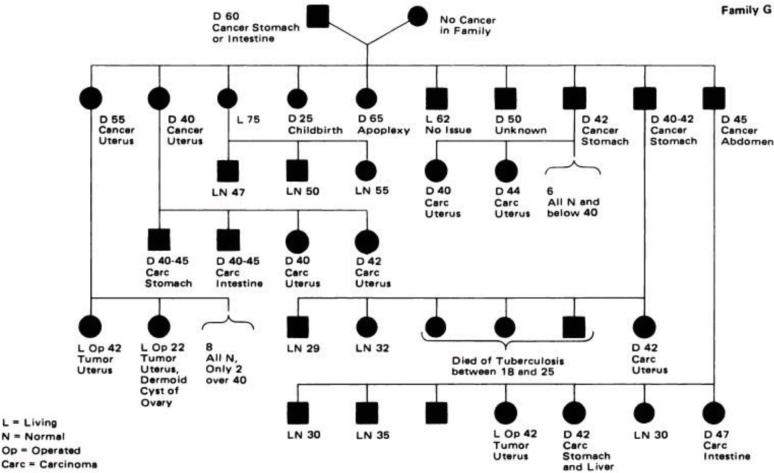
The Family G pedigree elaborated by Dr. Aldred Scott Warthin and published in 1913^49^. (D: dead).

Warthin became very interested in the cancer aggregation concerning Pauline’s familiar history, so he decided to conduct a meticulous investigation. After intense work, Warthin published this family pedigree in a seminal article around 110 years ago (Figure 2)^49^. It was entitled: *“Heredity with reference to carcinoma as shown by the study of the cases examined in the pathological laboratory of the University of Michigan, 1865–1913”*, where he concluded that:

*“A marked susceptibility to carcinoma exists in the case of certain family generations and family groups*. *The multiple occurrences of carcinoma in a family generation practically always mean its occurrence in a preceding generation”* ^49^.

For the first time, he raised attention to the dominant heritage present in this group he nominated “Family G”, as the oldest member came to the United States from Germany. This detailed study (one of the longest cancer family documentations ever recorded) revealed many generations affected by colonic, gastric, and endometrial cancers.

Afterward, he performed an audit of 3,600 cancer cases between 1895–1912 in the state hospital where he worked, revealing that 15% of them referred to family history of carcinoma, and the conclusion was that *“there was some influence of heredity on cancer”*.

In 1919, Pauline fastidiously improved and extended the original pedigree, correcting dates and missing descendants, marking the deaths, and adding new members as they came into the family, whether through birth or marriage^1^. It had been 25 years since they first started working together, and she was proud to hand her perfect paperwork over to Warthin^1^. The data Pauline provided about 150 relatives certainly helped Warthin to establish a clear pattern of cancer inheritance^1^. To acknowledge her contribution, he wrote: *“The writer had also an unusual opportunity of obtaining accurate information concerning various lines of descent in this family from an intelligent and cooperative member of the family”47.*

His conclusions about Family G were published in 1925, when he stated that gastrointestinal and gynecological tumors occurred at an early stage in successive generations (146 family members; 32% of them had cancers at the median age of 38 years)^47^. He also noted that the genetic transmission within these families was consistent with the Mendelian proposal of an autosomal dominant inheritance. Interestingly, he stated that “*his observations were not well accepted by surgical writers”* ^47^.

As an investigator deeply involved with hereditary cancer nowadays, Dr. James Michael Church considered that Warthin “*was in the right place at the right time to help develop this concept, although all this history may not have happened without the observations of his seamstress”* ^13^.

Pauline Gross was in fact the grand-grand aunt of a famous professional Canadian writer named Ami McKay, who published an amazing bibliographic book entitled: *“Before my Time, a memory of love and fate”,* originally named *Daughter of Family G*, where she beautifully told the sad history of her family and the impact of their genetic legacy on many generations^36,37^.

In this book, she affirms that Pauline did not want to get married due to the burden of her family’s genetics. Moreover, Pauline was, in part, frustrated by some errors committed by Dr. Warthin while constructing the family tree along the way^1,36,37^. By completing 25 years of this long journey together with Dr. Warthin by the summer of 1919, she corrected dates, missing descendants, and people coming into the family^1,36,37^. She was deeply proud of her work. A little time after that, she was diagnosed and operated on for an advanced uterus cancer, dying at the age of 46, in 1919.

Amy McKay wrote: *“My Pauline… is not a nameless seamstress who confessed her woes to a doctor and then vanished into thin air. She was an outspoken and courageous woman who came from a family who loved her. She was intuitive, yes, but also an astute observer of human nature and a stickler for detail. In many ways, for what was to unfold, Warthin needed Pauline far more than she needed him.” And continued: “A century after Pauline Gross’ death, her commitment to researching her family is helping to save their lives - and that is a remarkable legacy indeed”* ^1,11,36,37^.

This writer also produced a very insightful radio documentary for the Canadian Broadcasting Corporation (CBC), where she explored the family history and discussed the problems involved in the decision to undergo genetic testing (she tested positive for LS). This memoir *Daughter of Family G* won some awards in 2020^11^. (direct link: https://www.cbc.ca/radio/sunday/the-sunday-edition-for-september-29-2019-1.5299577/daughter-of-family-g-ami-mckay-s-2002-documentary-about-her-family-s-deadly-genetic-history-1.5299641).

Warthin’s findings were notable. His work was developed during an era that had not yet embraced the concept of heritable cancer predisposition. Some think that this American resistance against the association of heredity and cancer is due to the *“Do not delay”* ideology disseminated by the American Society of Cancer Control, defending the idea that early detection and surgery were the only means to fight this disease^40,51^. For this, he lamented *“the failure of the medical community to further study the role of heredity in the cause of neoplasms”* ^48^.

*Despite this compelling evidence, Warthin struggled to gain acceptance.* His ideas received notorious opposition from many Medical Societies, although being regarded as an internationally distinguished pathologist, his position as Editor of the Annals of Internal Medicine and as President of the American Association for Cancer Research^51^. Doctors used to consider hereditary aspects as part of a nosographical description, and the term “heredity” stood for a tendency for certain maladies to develop within a family^12^. The belief was that only the predisposition to the disease was inherited, not its features^40^.

After his death on May 23, 1931, his assistant Carl Vernon Weller continued the research although nobody knew the real cause of that unusual aggregation of cancers^17^. Using the same critical pathologic verification, he updated the kindred in 1936. Weller began a fourth evaluation of the kindred in 1955 but it was not completed due to his death the next year^28^. Thus, Pauline’s annotations and Warthin’s ideas could have been lost forever after Weller’s unexpected death in 1956. Fortunately, all findings were rediscovered in the 1960s by the American doctor Henry Thompson Lynch and the social worker Anne J. Krush, then working at Creighton University School of Medicine in Omaha, Nebraska^29^. Dr. Lynch himself wrote in a manuscript honoring all Dr. Warthin’s work regarding the possibility of inheritance in some families with cancer: “*Warthin can properly be called ‘the father of cancer genetics’, although the true value of his seminal work is not yet fully appreciated”* ^26^ *.* However, most authors did not agree and considered Henry Lynch to hold this distinction^33^.

### Henry Thompson Lynch (1928–2019): the real father of cancer genetics

**Figure 3:**
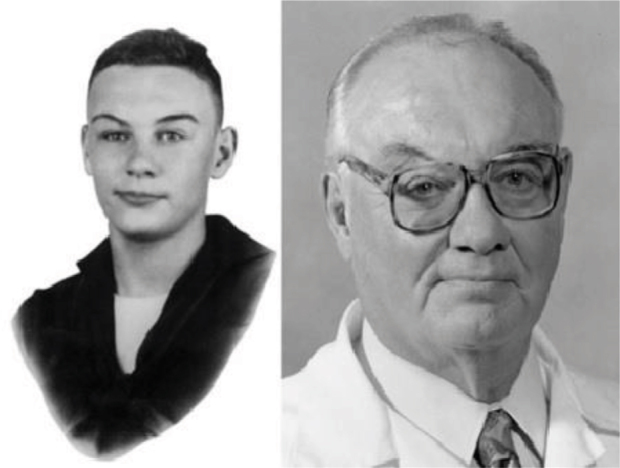
(Left) Henry Lynch as a young man^12^; undated photograph, probably taken in the 1940s. (Right) The father of hereditary colorectal cancer, Henry Thompson Lynch,1928–2019^6^.

Dr. Henry Thompson Lynch was born in Lawrence, Massachusetts (January 4, 1928) and grew up in a poor section of New York. He dropped out of school at 14 years of age and tricked his way into the United States Navy in 1944, using a cousin’s identification to make himself seem older than his 16 years old^12^. The subterfuge was discovered, but the Navy kept him on for the duration of the Second World War (Figure 3, left). Discharged from the Navy in 1946, he became a professional boxer, working predominantly in upper New York State and the San Francisco Bay area^12^. He was a large man and became a professional boxer under the name “*Hammerin’ Hank*”^12,35^. According to a close friend, a short engagement with boxing career was responsible for a scar over his right eyebrow, without affecting his neurological faculties^38^.

He completed internship at St. Mary Hospital in Evansville, Indiana, and medical residency in Internal Medicine at Nebraska College of Medicine. He then obtained PhD in Human Genetics at Texas University (Austin), where he also worked as Assistant Professor in MD Anderson Cancer Center^12^.

After Dr. Warthin’s publications, reports concerning the heritability of cancer were uncommon until Dr. Lynch described several groups with familial clusters of cancer during the 1960s, naming them *“Family Cancer Syndrome”* ^29,30^.

During his Medical Residency in Nebraska, Dr. Lynch (Figure 3, right) was intrigued by one drunk patient reporting many relatives with cancer (confirmed by his pedigree)^12^. He was diagnosed with familial adenomatous polyposis (FAP) by a gastroenterologist. However, his colon didn’t have any polyps. One year later, this patient developed adrenal cortical carcinoma at 44 years old^31,43^. Thus, he performed an epidemiological analysis of this group he named “Family N” (for Nebraska), discovering that they exhibited a CRC predisposition with dominant heritage, and many women had endometrial or ovary cancer. In 2000, a genetic evaluation demonstrated that Family N members had a germinative mutation in the *MSH2* gene.

**Figure 4:**
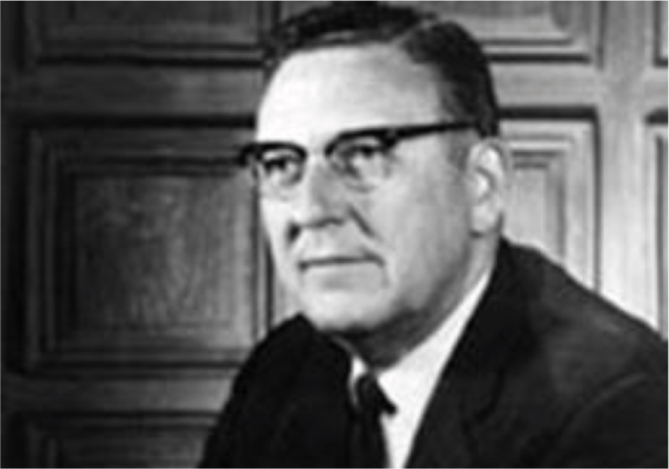
Dr. Adam James French (1912–1985), chairman at Pathology Department of Michigan University, the man who invited Lynch to update data from Family G. (Available from: https://www.iapcentral.org/home/hall-of-presidents/1966-1967-adam-james-french-usa)

In a fortuitous connection with the past at the University of Michigan, the Chairman of the Pathological Department called Adam James French (Figure 4) had succeeded Warthin, and turned to be responsible for the custody of all records and specimens related to Family G. Then, he invited Lynch to update this data and provided him with all the clinical, genealogical, and pathological information previously elaborated by Warthin and Pauline Gross^30^.

Subsequently, Lynch visited Germany to investigate family members who had not emigrated to the United States, discovering they also developed neoplasia in a similar pattern. After studying 650 Family G members, he published one iconic manuscript entitled “Cancer Family G Revisited” in 1971^28^.

In 2000, Warthin’s Family G finally got an answer when the mutation in the *MSH2* gene was reported in descendants (presence in the short arm of chromosome 2, where the *MSH2 gene* is located*)6,46*. Later on, an update developed in 2005 revealed 929 descendants known members^15^. Of this total, 40 underwent genetic sequencing and were found to have the same type of mutation^15^. Dr. Lynch wrote: *“There is probably no other instance in which one family has contributed so much understanding of an important genetic disease such as this”6.*

During Lynch’s public presentation about Family N in 1964 at the American Congress of Human Genetics, Dr. Margery Wayne Shaw (a geneticist from Michigan University at Ann Arbor) told him she was treating a family with similar patterns in Michigan^12,30^. Then, Lynch with his social worker Anne Krush, visited Ann Arbor to collect data about this family, and this group received the denomination of “Family M” (for Michigan)^12,30^.

This collaboration culminated with a publication about these two families (N-Nebraska and M-Michigan) in 1966^30^. They described similar cancer patterns in successive generations, with predisposition to CRC and other tumors at a young age, similar to Family G. This important aspect reinforced the evidence that cancers could have a genetic basis, showing that Lynch was facing a familiar aggregation consistent with an autosomal dominant inheritance pattern. Today, Family G is recognized as a LS group, having its last documentation in 1971, prior to the modern era of molecular diagnosis^28^.

Lynch studied families from the past, including that of Napoleon Bonaparte, whose cause of death in 1821 was carcinoma of the stomach, the same disease that killed his three sisters, brother, father, and grandfather^23^. Interestingly, one of Napoleon Bonaparte’s sisters, who died at the age of 44 probably as a result of gastric cancer, was called Pauline^23^!

During the early 1990s, the genetic cause of Family G was discovered, thanks to the efforts of Dr. Lynch and colleagues like Bert Vogelstein from Johns Hopkins University (a legendary cancer geneticist). The investigators found that defects in the so-called mismatch repair (MMR) genes were common in those families^25^.

### The nomenclature evolution

Historically, Dr. Henry Lynch described the familial aggregation of tumors (colorectal, stomach, endometrial) and suggested the name “Cancer Family Syndrome” (CFS) for this condition^6,29,30^. This term emerged from the finding that cancer may be more frequent in some families, requiring specific screening strategies. But during the 1970s and 1980s, there existed a lot of skepticism around the relation between cancer and genetics^12,31^. Opponents stated that those families were *“chance clusters of cancer”* or credited this chance to *“common environmental exposures”* or an eventual FAP misdiagnosis^12^. The contraposition of many medical societies forced him to finance his own work at the beginning, but the accumulation of evidence changed the acceptance of his work along time^12,27^.

Subsequently, the designation was adjusted to *Hereditary NonPolyposis Colorectal Cancer* (HNPCC) as suggested by Lynch himself^13,29^. With this, he wanted to differentiate this condition from other forms of hereditary CRC such as FAP. It is worthy mentioning that Lynch’s preference for this nomenclature was admirable, not only because both terms (CFS and HNPCC) helped to define the syndrome with a name and its clinical features but also because he never wanted to use his own name^13^. He still preferred HNPCC to designate families fulfilling the Amsterdam criteria. However, HNPCC was considered a misnomer at that time because it comprised hereditary cancers with distinct molecular basis, and these patients could also develop many adenomatous polyps^21,22^. This aspect is an irrefutable proof of his humility^13^.

The resurrection of the name LS occurred with the publication of its classification into types I and II^5-7^. This was considered necessary to separate families with a CRC predominance and others with full spectrum of cancers^21,22^. But it is not used anymore. It was only the clarification of the genetic basis unifying the diagnosis around a DNA-MMR germline mutation that finally determined the adoption of the nomenclature Lynch Syndrome (LS)^5,19^.

### The “Genetic ERA”

Advancements in medical genetics have experienced a great evolution over the last decades, culminating in the discovery of the genes responsible for LS. Propelled by the discovery of identifiers such as the microsatellite instability test (MSI) and immunohistochemistry (IHC), clinicians were able to optimize the diagnosis and treatment of affected patients^8,24,34^.

The syndrome accounts for approximately 5% of CRC and 2–4% of endometrial cancers, and those affected carry an 80% lifetime risk of developing CRC and other tumors at a young age. A key feature of LS-associated CRC is accelerated carcinogenesis, which was first described by Jass in 1994^20^.

The disorder is associated with prolonged survival, phenotype variations, predominance of synchronous (3 times higher) and metachronous cancers (5–7 times higher), early onset of carcinogenesis, predominance of proximal topography in the colon, and some specific histological features^20,32^. There is also a significantly increased frequency of gastric, hepatobiliary system, small bowel, urologic, and ovarian tumors. Neoplasias in other locations (glioblastomas, skin, pancreas, breast, prostate) are also overrepresented in LS patients^32,50^.

During the 1990s, investigations brought some light into the link between MSI and LS, following that MSI (repetitive sequence of nucleotides) was adopted to identify potential LS cancers, once these areas are susceptible to errors. Subsequently, mutations in other MMR genes were consecutively reported. The discovery of these genes facilitated the way for IHC testing and then to guide gene testing^50^. The establishment of molecular genetics is crucial to provide a differential diagnosis with other cancer syndromes^19,50^.

Because cancers develop as a consequence of mutations disturbing the function of DNA-MMR genes, CRC screening turns out to be fundamental, as they occur earlier than in normal subjects^10,45^. Based on the established cancer risks and specific genes, higher risks of gastrointestinal and extracolonic tumors are, in general, associated with *MLH1* and *MSH2* mutations, respectively^50^. Conversely, *MSH6* carriers lead to later onset tumors, while *PMS2* mutations have an overall lower risk for cancers.

The analysis of LS evolutional history clearly illustrates how the combination of clinical and scientific discoveries helps to integrate concepts. The limitations associated with the use of the Amsterdam criteria and Bethesda guidelines as indicators for DNA testing raised the evidence for the efficacy and cost-effectiveness of the universal screening strategy in LS^10^.

When used appropriately, a positive genetic test confirms the diagnosis, justifies surveillance of at-risk relatives, and may also help to define the best surgical and chemoprevention management^15^. Consequently, the diagnosis has significant public health implications for the patient and family. As genotype-phenotype relations are being consistently discovered, new individual strategies for screening, surveillance, and treatment must be developed to optimize follow-up^14,43^.

In Table 1, we present some known and most important characteristics of LS according to recent data reported by experts^18^.

**Table 1:** Common features associated with Lynch Syndrome18

What we know about LS?	Simple answer
Population prevalence	1: 279 people
Percentage of CRC burden	3% of CRCs and 3% of endometrial cancers
Diagnosis	Identification on molecular genetic testing of a germline heterozygous pathogenic variant in *MLH1, MSH2, MSH6, PMS2*, and EPCAM
Mutation frequencies	Highly population-specific
Mutation contributions for LS	*MLH1* (15–40%), *MSH2* (20–40%), *MSH6* (12–35%), *PMS2* (5–25%), and *EPCAM* (<10%)
Adenoma-cancer transformation	Carcinogenesis within a shorter time frame (2–3 years versus 6–10 years to evolve)
Pathological features	Diploid content, excess of mucinous and poorly differentiated cell types, villous components, and lymphocytic infiltration
Extent of colon surgery	Total colectomy will prevent metachronous cancers
Hysterectomy/oophorectomy	Should be offered to women with completed childbearing
Screening	Traditional screening programs were abandoned in favor of a universal screening approach

LS: Lynch Syndrome; CRC: colorectal cancer.

### Other historical investigators

HNPCC and LS terms were interchangeably used till 2006, when a classical article by the pathologist Jeremy Jass defined that LS should be used only for patients harboring MMR gene mutations and vertical transmission^19^. In the period between 1895 (Warthin’s seamstress statement) and the 1990s, little was added to the understanding of this condition. Fortunately, despite the denomination changes and all the controversies through the years, important investigators gave support to Warthin and Lynch’s ideas.

In 1973, Dr. Clement Richard Boland Jr. wrote a medical degree thesis entitled *“A Familial Cancer Syndrome”* ^4,6^. This manuscript was inspired by the intense curiosity about his own family’s long history of colon and other cancers^2,3^. The decision to conduct this and other investigations came after he lost his father due to synchronous colon cancer at 49 years old, when he was in the second year of medical school^2,3^. His grandfather had colon cancer at 27 and rectal cancer at age 46. The sister of his dad died at 27 also of colon cancer. According to his own words, *“there was something more than bad luck going on”* ^4,6^.

**Figure 5:**
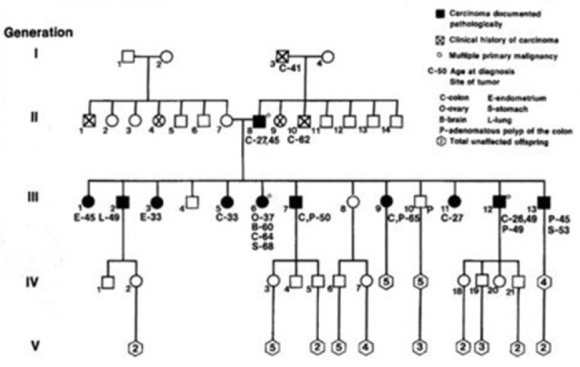
The family heredogram of Clement Richard Boland Jr.^3^

Then, he was convinced there was genetic cancer in his family (Figure 5). But at that time, there was very little understanding of the genetic basis of colon cancer, and very few families like his had been reported. When he was writing his thesis in 1972, he contacted Dr. Henry Lynch, who had just published an article in 1971 about Family G^28^. This contact probably inspired him to locate and study all the relatives he could find, obtain pathological reports, perform a literature search, and measure blood carcinoembryonic antigen (CEA) in all asymptomatic members^2,3^.

He had an outstanding academic career. The focus on the genetic causes of colon and familial cancer syndromes turned him into one of the first gastroenterologists to explore MSI in cancer. Running a major Gastrointestinal Cancer Laboratory at Baylor University in Dallas, he developed the first in vitro models to study the basic biology of LS. Just a few years later, he cloned the gene that caused cancer in his father.

Progressively, doubts about the existence of a hereditary nonpolyposis syndrome started to fade with the great help of Dr. Hans Vasen from the Netherlands, who had an important role in the refinement of clinical diagnostic criteria (the famous Amsterdam Criteria)^44^. Since 1985, he had worked as Medical Director of the Netherlands Foundation for the Detection of Hereditary Tumors. He established a large register of families with inherited predisposition to cancer, developed surveillance protocols, and helped to translate molecular genetic results into clinical practice.

Another notable investigator is Dr. Heikki J. Järvinen from the University of Helsinki, who developed research in the genetic background and histological features of LS. He also studied the feasibility of molecular screening and potential oncogenes associated with MSI.

### Henry T. Lynch’s legacy

All the prestigious activities of Dr. Henry Lynch as a doctor, scientist, and professor brought him international recognition as the *“Father of Hereditary Colorectal Cancer”*. Working as a Professor at Creighton University since 1967, he created the important Hereditary Cancer Center for prevention in 1984, where he dedicated many years of his luminous life. It is important to emphasize that he was designated Honorary President of the Kamie K. Preston Hereditary Cancer Foundation^35^.

**Figure 6:**
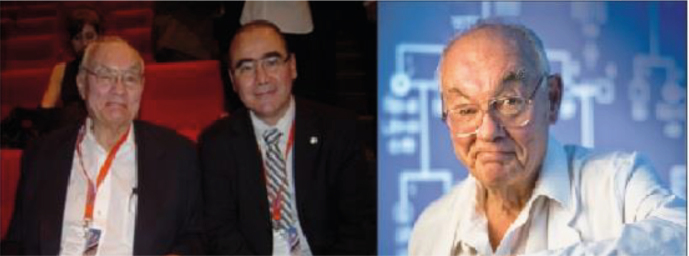
(Left) Henry Thompson Lynch and Fabio Guilherme Campos at a medical meeting in Buenos Aires (Argentina, 2010 – personal archives). (Right) Henry Lynch in 2016 (Available from: https://www.thelancet.com/cms/attachment/094e10ed-7d07-45eb-8408-b6596083bea2/fx1.jpg)

Professor Henry Lynch made many friends among members of the Brazilian Society of Colorectal Surgeons (Figure 6). In July 2017, two of the present authors (CARM and FGC) had already published a homage to him, available on the Brazilian Society of Coloproctology homepage, where we stated so many of his activities in life^35^.

Thus, the present manuscript is nothing more than another tribute to his remarkable life as a magnificent investigator and healthcare provider. Among his many qualities, it is worth mentioning that he was able to understand the origin of many cancers by simply taking a good family history. We now know that 15–20% of patients with colorectal tumors refer to a previous diagnosis in one or more parents, siblings, or offspring^31^.

Moreover, he manifested a great capacity to be persistent in evaluating a problem he had to confront and also to make clinical observations on individual patients with unusual patterns of disease^41^.

Henry Lynch died due to heart failure in Omaha in 2019, at age 91, seven years after the passing of his wife Jane^33^. Although he was not the pioneer in recognizing different inheritance patterns, we must render him the merits for rediscovering this issue and for defending the concept of hereditary CRC even against furious opposition^12,27^. His perseverance was fundamental in convincing the medical community about the importance of hereditary CRC and family prevention.

Having the eponym Lynch Syndrome rendering an obvious and deserved homage to his name clearly reflects the wide recognition of all his productive work to identify cancer syndromes, patterns of inheritance, and cancer detection. He also discovered the Mendelian patterns of inheritance in breast and ovarian cancers, contributing to the discovery of the *BRCA1* and *BRCA2* mutations.

Indeed, he received many awards from important medical societies during his long career. For good, the great dimension of his legacy lies in the fact that his efforts and dedication helped us understand the relation between cancer and genetics. We are sure that his numerous contributions will influence medical decisions and generations for many years to come.

Considering the information dimension we have conquered over the past 30 years, we are sure there is still a long way to go in our understanding of LS. The amazing history we redeemed here makes us feel encouraged by what is expected in the future. As a message, the remarkable contributions of Pauline’s intuition, Warthin’s perseverance, and Lynch’s consistency must never be forgotten by those who already have or will still benefit from all the knowledge they provided us.
